# Association of Uremic Toxins and Inflammatory Markers with Physical Performance in Dialysis Patients

**DOI:** 10.3390/toxins10100403

**Published:** 2018-10-01

**Authors:** Maja Pajek, Alexander Jerman, Joško Osredkar, Jadranka Buturović Ponikvar, Jernej Pajek

**Affiliations:** 1 Faculty of Sport, University of Ljubljana, Gortanova 22, 1000 Ljubljana, Slovenia; maja.pajek@fsp.uni-lj.si; 2 Department of Nephrology, University Medical Centre Ljubljana, Zaloška 2, 1525 Ljubljana, Slovenia; alexander.jerman@kclj.si (A.J.); jadranka.buturovic@kclj.si (J.B.P.); 3 Clinical Institute for Clinical Chemistry and Biochemistry, University Medical Centre Ljubljana, Zaloška 2, 1525 Ljubljana, Slovenia; josko.osredkar@kclj.si

**Keywords:** hemodialysis, 6-minute walk test, sit-to-stand test, handgrip strength, Human Activity Profile, sarcopenia, muscle, asymmetric dimethyl-arginine, C-reactive protein, motor ability

## Abstract

Association of higher serum levels of uremic toxins and inflammatory markers with poorer physical performance is understudied. We measured the six-minute walk test (6MWT), 10 repetition sit-to-stand test (STS-10), handgrip strength (HGS), and Human Activity Profile (HAP) questionnaire score in 90 prevalent hemodialysis patents, with low comorbidity to reduce the potential confounding of concomitant disease. Midweek pre-dialysis serum levels of asymmetric dimethyl-arginine (ADMA), β2-microglobulin (B2M), high-sensitivity C-reactive protein (hs-CRP), indoxyl sulfate (IS), insulin-like growth factor 1 (IGF-1), interleukin 6 (IL-6), myostatin, and urea were analyzed as predictor parameters of physical performance measures in adjusted models. Serum levels of most measured toxins were not significantly related to performance, except for ADMA, which was significantly related to poorer performance in the STS-10 test (B = 0.11 ± 0.03 s, *p* < 0.01). Higher hs-CRP was associated with poorer results in the 6MWT (B = −2.6 ± 0.97 m, *p* < 0.01) and a lower HAP score (B = −0.36 ± 0.14, *p* = 0.01). There were no other significant associations found. We conclude that inflammation may be a more important pathway to physical impediment than uremic toxemia. This suggests that there is a large physical rehabilitation potential in non-inflamed uremic patients.

## 1. Introduction

Poor physical performance and frailty predict a high risk of mortality, cardiovascular events, and hospitalizations in dialysis patients [1,2]. Motor performance in dialysis patients is reduced and greatly affected by some clinical modifiable and non-modifiable anthropometric factors [3,4]. Decreased physical performance is a consequence of multiple factors, such as comorbidity, protein energy wasting, abnormal body composition, inadequate nutrition, and a sedentary lifestyle [5]. Uremia-induced alterations of increased energy expenditure, inflammation, acidemia, and hyper-metabolism may play a part as well [6]. So far, the relative proportions to which various components of uremic milieu add to poor physical performance, morbidity, and mortality are unknown.

Uremia is defined by a state of elevated serum concentrations of various uremic toxins [7]. Uremic toxins are categorized as free water-soluble, low-weight, middle-weight, or protein-bound molecules. Overall, there is a poor relationship between serum toxin concentrations and either the estimated glomerular filtration rate in chronic kidney disease (CKD) stage 2–5 patients [8] or the urea purification adequacy parameter Kt/V in dialysis-dependent patients; predialysis serum levels of uremic toxins are predominantly associated with protein intake and residual renal function [9].

In this work, we set out to analyze the association of serum uremic toxin levels with reduced physical performance of dialysis patients. We hypothesized that patients with higher concentrations of uremic toxins might suffer a greater loss of motor abilities. As patients with CKD have many comorbid diseases that may interfere with motor abilities, our study aimed to identify associations of five representative uremic toxins with physical performance measures in a sample of low-comorbid dialysis patients. We also analyzed two serum markers of inflammation—interleukin 6 (IL-6) and high-sensitivity C-reactive protein (hs-CRP)—to account for the effect of inflammation, which is an important part of uremic milieu muscle catabolism [10]. Additionally, two metabolic mediators associated with muscle mass, insulin-like growth factor 1 (IGF-1), and myostatin were analyzed as well.

## 2. Results

Ninety prevalent hemodialysis patients fulfilled the inclusion criteria. They were all treated with high-flux dialyzers without reuse, and 52% of them were treated with on-line hemodiafiltration. The baseline demographic characteristics are shown in the Table 1.

### 2.1. Six-Minute Walk Test

The analysis of predictive parameters of the six-minute walk test (6MWT) test included age, sex, patient body height, fat tissue index, and serum levels of the selected uremic toxins. Certain anthropometric factors adjusted here—age, height, and fat tissue index (FTI)—were previously shown to be predictive for the results of the 6MWT [4]. The estimates for individual uremic toxins and other markers in the linear adjusted model and their variation estimates are shown in Table 2. The only parameter significantly negatively associated with performance was hs-CRP.

### 2.2. Ten-Repetition Sit-to-Stand Test

We have previously shown that age, body height, and lean tissue index (LTI) predict 10-repetition sit-to-stand (STS-10) test performance [11]. Therefore, we adjusted for these factors in the models incorporating individual uremic toxins and other markers as predictors of STS-10 results. Asymmetric dimethyl-arginine (ADMA) was the only toxin significantly associated with longer time to perform this exercise (see Table 3).

### 2.3. Hand Grip Strength

It was previously shown that sex, age, body height, and wrist width are non-modifiable predictors of hand grip strength (HGS), whereas LTI and FTI are potentially modifiable predictors [3]. Table 4 shows the results of models, including the aforementioned predictors, and each of selected uremic toxins and other markers. No significant associations with uremic toxins and metabolic or inflammation markers were found in the adjusted analyses.

### 2.4. Human Activity Profile Questionnaire Results for Habitual Daily Activity

Habitual daily physical activity, estimated with the HAP adjusted activity score (AAS) is known to be associated with age and LTI [3], so the association of each of the uremic toxins and other selected markers was adjusted for these two covariates. The results for parameter estimates are shown in the Table 5. Again, only hs-CRP was significantly associated with a lower score.

Finally, in Table 6 we summarize the predictive values of all significant predictors in above-mentioned analyses. Partial eta squared coefficients show that the proportion of explained variability in the examined physical performance measures is in the rage of 7.2% to 14.3%. The most consistent association with physical performance was found for inflammatory marker hs-CRP, which significantly predicted performance in the 6MWT and HAP questionnaire. Uremic toxins and metabolic mediators were not predictive of physical performance, except for ADMA in STS-10 performance.

## 3. Discussion

In this work, we examined the hypothesis that higher serum levels of uremic toxins predict worse physical performance in end-stage renal disease patients. We were able to recruit low-comorbid group of 90 prevalent hemodialysis patients, and assess four different measures of physical performance. We selected representative uremic toxins from three classes according to molecular weights and protein binding properties [7,12]. Previously reported potential effects of uremic toxins on the osteo-muscular and nervous systems—the two systems that take part in the control of movement and motor tasks—were taken into consideration in the process of selecting candidate toxins, as revealed in the methods section. Overall, the results of our study did not confirm our hypothesis. We were not able to find a consistent association between uremic toxins and physical performance, with the exception of ADMA, which was associated with a longer time to complete the STS-10 test. In contrast to the overall impact of various uremic toxins, our data suggest that inflammation is a more important factor of physical performance loss. Specifically, higher values of hs-CRP predicted poorer performance in the 6MWT and habitual daily activity, as measured by the HAP questionnaire.

ADMA is a recognized toxin with a role in cardiovascular and renal disease, but exact mechanisms for its toxicity are unknown. It is an endogenous inhibitor of nitric oxide (NO) synthesis [13], and increased levels are associated with decreased endothelial function; in non-diabetics, levels are also correlated with insulin resistance [14]. There is some laboratory evidence that elevated levels of ADMA may contribute to skeletal muscle proteolysis [15], which may also be a mechanism for reduced physical capacity in the STS-10 performance found in our study. Association of ADMA with the loss of physical fitness was confirmed for patients with heart failure [16] and in non-uremic elderly persons [17]. Currently no specific, clinically applicable means to lower the ADMA level is available for renal failure patients.

Chronic low-level inflammation is associated with poor cardiovascular outcomes [18,19] and cardiovascular morbidity [20,21]. If this inflammation presents concomitantly with poor physical activity, it bears a high risk of mortality [22]. Inflammation was linked to muscle atrophy in the patient cohort with end-stage renal disease [23]. Impaired intracellular insulin signaling, involving insulin and IGF-1, might be the causal link between inflammation and muscle protein degradation. Increase in IL-6 is linked to decreased levels of insulin receptor substrate 1 (IRS-1), and this leads to increased proteolysis [24]. Inflammation activates myostatin, which negatively regulates muscle growth via activation of protein degradation with the ubiquitin–proteasome system [25,26]. Additionally, inflammation also causes endothelial dysfunction (ED), which is aggravated by the action of ADMA through inhibition of NO synthesis (see Materials and methods). ED mediates atherosclerosis [27], which may have long-term deleterious effects. In incident and prevalent hemodialysis patients, inflammation is linked to adiposity and increased mortality risk [28]. Importantly, recent work from Delgado and coworkers suggest that larger amount of visceral fat is associated with increased inflammation, while peripheral fat is associated with improved markers of nutrition [29]. Our results have shown that for the six-minute walk distance, there was an independent negative association of hs-CRP with walked distance in the models, with or without adjustment for the fat tissue index, which may point to an effect of inflammation beyond the contribution from visceral fat.

Our study has some limitations. Since this was an observational study, we are unable to prove any causal relationship. We performed a number of statistical models, and therefore introduced the possibility for a raised alpha error. Also, if the number of participants was larger, we could perhaps confirm as significant more subtle associations; however, the number of participants was comparable to previous studies of motor performance in the dialysis population [30]. There is a known intra-patient variability of some uremic toxins, especially for ADMA and IS time-averaged concentration would be a better representation of exposure than a single midweek pre-dialysis value [31]. The strengths of our study include the recruitment of patients with low comorbidity, thus limiting the confounding effect of comorbidities on physical performance. In addition, based on our previous work, we were able to control for important non-modifiable anthropometric covariates that were found to affect performance. We also included markers of inflammation and crucial muscle metabolic mediators (IGF-1, myostatin) in our analysis, thus extending the analysis on these two pathways of a possible performance loss.

In conclusion, our analysis of selected uremic toxins in low-comorbidity hemodialysis patients revealed no consistent associations with physical performance in four major motor ability tests. We could only find a significant association of higher ADMA levels with poorer lower extremity function during the STS-10 test. More consistent was a predictive role of inflammation, expressed as elevated hs-CRP levels, for a loss of 6MWT distance and habitual activity in everyday life. This finding suggests that regular physical training and activity may have a large rehabilitation potential, possibly to the level of healthy controls in non-inflamed, low-comorbid, end-stage renal disease patients.

## 4. Materials and Methods

### 4.1. Study Design and Participants

This was a cross-sectional study recruiting a sample of prevalent hemodialysis patients from 10 outpatient dialysis units in Slovenia. Measurements were done in the period from July to December 2014. Main outcome measures were the results of key functional performance tests covering the motor abilities of the upper extremities (HGS), the lower extremities (STS-10), submaximal aerobic performance (6MWT), and the result of the Human Activity Profile questionnaire (HAP) to assess the level of physical abilities and activities in the home environment. Subjects were invited to participate if older than 18 years, able to walk with or without additional support, and if they voluntarily signed the informed consent for participation. Exclusion criteria contained acute disease in the last four weeks before the start of the study, active malignant disease or chronic infection, consequences of cerebrovascular accident, heart failure of New York Heart Association (NYHA) stage 3–4, symptomatic angina pectoris (Canadian Cardiovascular Society) stage 2–4, chronic obstructive pulmonary disease stage 3–4, decompensated liver cirrhosis, symptomatic peripheral arterial obstructive disease, painful degenerative or inflammatory arthropathy with current use of analgesic therapy, and symptomatic psychiatric disease. The study was approved by the Slovenian Medical Ethics Committee (document No. 125/05/14). Investigations were carried out following the rules of the Declaration of Helsinki of 1975, revised in 2008. All participants gave informed consent to inclusion in the study.

### 4.2. Research Protocol

The exact description of the research protocol and measurements was given previously [4,11,32]. In short, physical performance tests were performed in the afternoon hours on non-dialysis days (mean time lag from the last dialysis session was 25 ± 6 h). Application of HAP Questionnaire was followed by anthropometric measurements (instruments by SiberHegner, Zurich, Switzerland), vital signs recordings, and bioimpedance body composition analysis (Body Composition Monitor, Fresenius Medical Care, Bad Homburg, Germany). Here, estimated lean and fat mass in kg were normalized to squared body height (in m^2^) to give the LTI and FTI. Then, physical performance tests were done. Additional medical information on comorbidities and most recent routine blood test laboratory values were obtained from dialysis centers and attending physicians. Concentrations of uremic toxins were measured from the serum midweek pre-dialysis samples taken at participating dialysis centers, and transported immediately to the central laboratory within the two-hour period in the week immediately subsequent to physical performance measurements.

The 6MWT was performed on a 30 m track according to the guidelines [33], with the traveled distance measured to the nearest meter. A Jamar hand dynamometer (Sammons Preston, Warrenville, IL, United States) was used to assess HGS, engaging both hands three times, with the best value of all attempts was taken as a result (in kg units). The STS-10 time (in seconds) was measured as the time needed to perform rises from the chair of a standard height to the full leg extension and back to sitting position 10 times in a single attempt. HAP questionnaire is used to assess the level of physical activity, with the result reported as the adjusted activity score (AAS), on a scale of 1–94 points (larger values designate higher level of ability in everyday life) [32,34,35]. Previously, the HAP questionnaire was recognized as the single best substitute for quantitative measurement of habitual physical activity in hemodialysis patients [34].

### 4.3. Rationale for the Choice of Uremic Toxins and Measurement Methods

We measured serum levels of urea, asymmetric-dimethylarginine (ADMA), β2-microglobulin (B2M), indoxyl sulfate (IS), myostatin, interleukin-6 (IL-6), high-sensitivity C-reactive protein (hs-CRP), and insulin-like growth factor 1 (IGF-1). We used reagent kits based on ELISA or chemiluminescent technology, and routine laboratory methods for the measurement serum levels of hs-CRP. All procedures followed the instructions from manufacturers. With values above the linearity range, we diluted samples following instructions in the manufacturer kit. For the values below the detection limit, we expressed the results as a lower limit of detection divided by the square root of two. We present details for these methods in Table 7.

Urea is a prototype of small (60 Da) water-soluble uremic toxins, giving the name to the uremic syndrome. Because of a complex metabolism, it is not a reliable marker of kidney function loss. However, an indirect toxicity has been established, and some evidence supports direct toxicity of urea [36].

ADMA is a small water-soluble molecule (202 Da), similar to urea. It belongs to a group of guanidines, known neurotoxins that have a significantly larger distribution volume than urea. This may result in decreased removal with conventional dialysis techniques. In-vitro studies show that guanidines have a pro-inflammatory effect at uremic concentrations. ADMA is an inhibitor of nitric oxide synthase, with a direct effect on cellular dysfunction [37]. It was correlated with increased intima-media thickness in dialysis patients [38], vascular damage, proteinuria, amyloidosis [39], cardiovascular outcome, and all-cause mortality [40]. Importantly, published data suggests that the complex metabolism of ADMA can be modified with interventions targeting the enzyme dimethylarginine–dimethylaminohydrolase, achieving a lower concentration and change in vascular status [38].

B2M is a middle-weight protein (11.8 kDa), included here as it is often used as a marker of other retention solutes of the same size class. It is implicated in the genesis of uremic amyloid disease [41] in chronic hemodialysis patients, as well as their increased total [42] and infectious mortality [43]. Longer dialysis treatment with a high-flux membrane decreases predialysis β2-microglobulin levels, which is further improved by adding hemofiltration [38]. Part of β2-microglobulin’s clearance might be associated with adsorption to the dialyzer membrane [44,45].

IS is a protein-bound molecule (213 Da), and as such, it is representative of a group of relatively small protein-bound uremic toxins that are not easily removed with low- or high-flux dialysis membranes. It is suspected that IS conveys its in-vitro and in-vivo effect via a pro-oxidant mechanism, one of them being induction of ROS in endothelial cells [46]. It has been found to be associated with endothelial dysfunction, atherosclerosis, vascular calcification, smooth muscle proliferation, and progression of CKD due to pro-inflammatory and pro-fibrotic mechanisms [47]. Furthermore, it has also been associated with higher mortality in CKD patients [48].

Myostatin is a member of the transforming growth factors beta superfamily, and is negatively associated with muscle growth and development. Its molecular mass is 25 kDa, and high-flux dialyzer membranes may partially remove it. Serum levels are increased in uremic patients and in patients with heart failure [49,50]. Myostatin is probably directly involved in cardiac cachexia in heart failure patients, and its direct inhibition positively affects skeletal muscle mass [50]. Importantly, patients with advanced heart failure were excluded from our study (see above) to allow for evaluation of isolated myostatin association with muscle performance in uremia.

IL-6 is a middle molecular weight protein of 24.5 kDa [47]. It is produced by many immune, fat, and peripheral muscle cells. It has both pro- and anti-inflammatory action, but the former is more prominent. It has been shown to be a reliable prognostic factor for mortality in CKD patients [49], as it induces catabolism, lipolysis, and insulin resistance. Although it is partly adsorbed to a high-flux dialyzer membrane [45], this clearance is probably too small to affect long-term mortality or morbidity [44]. Several studies have shown small water-soluble guanidines to be responsible for the increased generation of IL-6 [51] and tumor necrosis factor alpha [52]. IL-6 was included in this study to represent the group of uremic toxins with inflammatory action. As inflammation may be one of the key pathways for induction of muscle catabolism and perhaps poor performance, hs-CRP was measured as well. C-reactive protein (CRP) is a marker of inflammation, produced in the liver cells under the influence of IL-6 and possibly other factors. CRP is very stable, the measurements are well standardized, and it is used widely for the assessment of inflammation. It is a well-known cardiovascular disease risk factor [53]. It was added to our analysis to mitigate the examination of inflammation as a possible pathway for reduced physical performance.

IGF-1 is a middle-weight protein (7.7 kDa). Its impairment by inflammatory factors is involved in muscle proteolysis [49], but the serum concentrations are elevated in CKD patients [7]. It has been also been associated with malnutrition [54]. It was included here as a possible mediator of both inflammatory and metabolic derangements of uremic milieu.

### 4.4. Statistical Analyses

Baseline patient characteristics were calculated as means and their standard deviations for continuous variables, and frequencies for categorical variables. We performed a series of general linear models, with individual physical performance measures as dependent and uremic toxin levels as predictor variables. In each model, besides standard parameters, partial eta squared was calculated, which gives the proportion of variance in the dependent variable explained by each independent predictor variable. For each uremic toxin, a separate model—including selected demographic and non-modifiable anthropometric variables (such as height)—was calculated. We only adjusted for those case-mix and non-modifiable anthropometric variables that we previously showed to be significantly associated with physical performance test results analyzed here (1st model) [3,4,11]. The body composition indices LTI and FTI were considered to be modifiable parameters that may lie in the causal pathway from uremic toxin level to the physical performance measurement. Therefore, in an exploratory analysis, we constructed a separate model (2nd model) incorporating one or both of these indices in cases where previous research showed that one or both of them may associate significantly with a specific physical performance measure.

We performed adjusted analyses by entering all independent variables of interest simultaneously. No stepwise methods were executed. Probability levels of <0.05 were considered statistically significant. Analyses were conducted using the IBM SPSS statistics (v. 22, IBM Corporation, Armonk, NY, USA) and SAS Studio (Rel. 3.6, Basic edition, SAS Institute Inc., Cary, NC, USA).

## Figures and Tables

**Table 1:** Patient demographic, body composition, and clinical characteristics (*n* = 90).

Parameter	Mean	Std. dev. or IQR
Female	29/90 (32%)	/
Age (years)	55.2	16.0
Patient height (cm)	168.3	9.1
Patient weight (kg)	74.4	15.5
Body mass index (kg/m^2^)	26.1	4.1
Fat tissue index (km/m^2^)	12.0	4.7
Lean tissue index (kg/m^2^)	13.6	2.7
Serum creatinine (µmol/L)	877	232
Serum albumin (g/L)	41.2	3.6
Davies comorbidity grade 0/1/2 (*n* (%))	47 (52.2)/ 37 (41.1)/ 6 (6.7)	/
Dialysis vintage (years)	4.6	1.7–11.5
Weekly dialysis time (h)	14	12–15
Hemoglobin (g/L)	119	12
Serum phosphate (mmol/L)	1.6	0.4
Serum intact PTH (pmol/L)	289	128–574

n: number of patients. Std. dev.: standard deviation. IQR: inter-quartile range. /: not applicable.

**Table 2:** The serum toxin estimates for prediction of a six-minute walk test (6MWT) result (distance in meters). The R^2^ coefficient and *p*-value for the selected significant model is shown just below.

Parameter	1st Model				2nd Model			
	B (m)	Std. err.	Sig.	Partial Eta Squared	B (m)	Std. err.	Sig.	Partial Eta Squared
ADMA	−0.21	0.50	0.67	<0.01	−0.25	0.47	0.60	<0.01
B2M	−2.90	2.20	0.19	0.02	−2.80	2.07	0.18	0.02
hs-CRP †	−3.096	1.04	<0.01	0.10	−2.63	0.97	<0.01	0.08
IGF-1	−0.00	0.00	0.54	0.01	0.00	0.00	0.92	<0.01
IL-6	−0.01	0.00	0.14	0.03	−0.01	0.00	0.19	0.02
IS	−0.45	4.82	0.93	<0.01	−0.36	4.48	0.94	<0.01
Myostatin	−0.65	0.52	0.21	0.02	−0.54	0.49	0.27	0.01
Urea	0.812	1.31	0.54	0.01	0.39	1.24	0.75	<0.01
Fat tissue index	/	/	/	/	−6.55	1.74	<0.01	0.14

1st model was adjusted for age, sex, body height; 2nd model was adjusted also for fat tissue index. ADMA: asymmetric dimethyl-arginine. B2M: β2-microglobulin. hs-CRP: high-sensitivity C-reactive protein. IGF-1: insulin-like growth factor 1. IL-6: interleukin 6. IS: indoxyl sulfate. B: B coefficient in analysis of variance general linear model. Std. err.: standard error. Sig.: statistical significance. †: 1st model: R^2^ = 0.554, *p* < 0.0001, 2nd model: R^2^ = 0.618, *p* < 0.0001.

**Table 3:** The serum toxin estimates for the 10-repetition sit-to-stand (STS-10) test (time in seconds). The R^2^ coefficient and *p*-value for the selected significant model is shown just below.

Parameter	1st Model				2nd Model			
	B (s)	Std. err.	Sig.	Partial Eta Squared	B (s)	Std. err.	Sig.	Partial Eta Squared
ADMA †	0.11	0.03	<0.01	0.11	0.11	0.03	<0.01	0.14
B2M	−0.13	0.16	0.41	0.01	−0.08	0.15	0.59	<0.01
hs-CRP	0.11	0.08	0.15	0.03	0.11	0.07	0.13	0.03
IGF-1	0.00	0.00	0.43	0.01	0.00	0.00	0.78	<0.01
IL-6	0.00	0.00	0.68	<0.01	0.00	0.00	0.36	0.01
IS	−0.19	0.34	0.57	0.01	−0.19	0.32	0.56	0.01
Myostatin	−0.01	0.04	0.81	<0.01	−0.01	0.04	0.75	<0.01
Urea	−0.07	0.09	0.47	0.01	−0.00	0.09	0.97	<0.01
Lean tissue index	/	/	/	/	−0.84	0.25	<0.01	0.14

1st model was adjusted for age and body height, 2nd model was also adjusted for lean tissue index (LTI). B: B coefficient in analysis of variance general linear model. Std. err.: standard error. Sig.: statistical significance. †: 1st model: R^2^ = 0.5, *p* < 0.0001; 2nd model: R^2^ = 0.57, *p* < 0.0001.

**Table 4:** The serum toxin estimates for hand grip strength (HGS, force of grip in kilograms). The R^2^ coefficient and *p*-value for the selected significant model is shown just below.

Parameter	1st Model †				2nd Model †			
	B (kg)	Std. err.	Sig.	Partial Eta Squared	B (kg)	Std. err.	Sig.	Partial Eta Squared
ADMA	0.04	0.04	0.29	0.02	0.03	0.04	0.47	0.01
B2M	0.13	0.17	0.46	0.01	0.06	0.17	0.74	<0.01
hs-CRP	−0.05	0.09	0.55	0.01	−0.06	0.08	0.48	0.01
IGF-1	0.00	0.00	0.82	<0.01	0.00	0.00	0.82	<0.01
IL-6	−0.00	0.00	0.79	<0.01	0.00	0.00	0.28	0.02
IS	0.41	0.37	0.27	0.02	0.47	0.36	0.19	0.02
Myostatin	0.05	0.04	0.24	0.02	0.03	0.04	0.47	0.01
Urea	0.20	0.10	0.05	0.05	0.17	0.10	0.10	0.03
Lean tissue index *	/	/	/	/	0.61	0.31	0.05	0.04
Fat tissue index **	/	/	/	/	0.15	0.15	0.33	0.01

1st model was adjusted for age, sex, wrist width, and body height, 2nd model was adjusted also for fat tissue index (FTI) and LTI. B: B coefficient in analysis of variance general linear model. Std. err.: standard error. Sig.: statistical significance. †: 1st model: R^2^ = 0.67, *p* < 0.0001, 2nd model: R^2^ = 0.71, *p* < 0.0001. *: only entered in the second model. **: only entered in the second model.

**Table 5:** The serum toxin estimates for Human Activity Profile (HAP) adjusted activity score (AAS).

Predictor Parameter	1st Model				2nd Model			
	B (AAS)	Std. err.	Sig.	Partial Eta Squared	B (AAS)	Std. err.	Sig.	Partial Eta Squared
ADMA	−0.07	0.073	0.34	0.01	−0.07	0.07	0.29	0.02
B2M	−0.03	0.34	0.92	<0.01	−0.13	0.30	0.67	<0.01
hs-CRP †	−0.35	0.16	0.03	0.07	−0.36	0.14	0.01	0.07
IGF-1	−0.00	0.00	0.12	0.03	−0.00	0.00	0.28	0.02
IL-6	0.00	0.00	0.58	<0.01	0.00	0.00	0.94	<0.01
IS	−0.12	0.73	0.87	<0.01	−0.07	0.65	0.91	<0.01
Myostatin	−0.03	0.08	0.72	<0.01	−0.06	0.07	0.41	0.01
Urea	0.13	0.20	0.52	0.01	−0.05	0.18	0.78	<0.01
Lean tissue index	/	/	/	/	2.12	0.44	<0.01	0.21

1st model was adjusted for age, 2nd model was adjusted for LTI. B: B coefficient in analysis of variance general linear model. Std. err.: standard error. Sig.: statistical significance. †: 1st model: R^2^ = 0.33, *p* < 0.0001. 2nd model: R^2^ = 0.48, *p* < 0.0001.

**Table 6:** Summary of significant associations of inflammatory markers and uremic toxins with physical performance measures found in this study.

Test	Parameter	B	Std. err.	Sig.	Partial Eta Squared
6MWT	hs-CRP	−2.63 m	0.97	0.0037	0.08
STS-10	ADMA	0.11 s	0.03	0.0009	0.14
HAP	hs-CRP	−0.36	0.14	0.0134	0.07

B: B coefficient in analysis of variance general linear model. Std. err.: standard error. Sig.: statistical significance.

**Table 7:** Reagent kits used with quality control data.

Parameter	Producer	Range	Sensitivity	Intra-Assay	Inter-Assay
ADMA	Antibodies-online (Atlanta, GA, USA)	7.8–500 µg/L	1.95 µg/L	<8%	<10%
B2M	Antibodies-online (Atlanta, GA, USA)	0.025–10 mg/L	0.025 mg/L	3.2%	8.7%
hs-CRP	Roche (Basel, Switzerland)	0.15–20 mg/L	0.15 mg/L	0.4%	1.6%
IGF-1	Abcam (Cambridge, UK)	0.1–30 µg/L	0.2 µg/L	<10%	<12%
IL-6	Abcam (Cambridge, UK)	1.56–50 pg/mL	0.8 pg/mL	4.4%	8.7%
IS	Antibodies-online (Atlanta, GA, USA)	1.0–25 µg/L	0.1 µg/L	<10%	12%
Myostatin	Immunodiagnostik AG (Bensheim, Germany)	0.6–500 µg/L	0.37 µg/L	10.4%	12.2%
Urea	Siemens Healthineers (Erlangen, Germany)	1.8–53.6 mmol/L	0.36 mmol/L	1.0%	1.5%

ADMA: Asymmetric-dimethylarginine; B2M: β2-Microglobulin; IL-6: interleukin 6; IS: indoxyl sulfate; IGF-1: insulin-like growth factor 1; hs-CRP: high-sensitivity C-reactive protein.
